# Vertical Heterophoria and Postural Control in Nonspecific Chronic Low Back Pain

**DOI:** 10.1371/journal.pone.0018110

**Published:** 2011-03-30

**Authors:** Eric Matheron, Zoï Kapoula

**Affiliations:** 1 Groupe IRIS CNRS/FRE 3375: Service d'Ophtalmologie-ORL-Stomatologie, Hôpital Européen Georges Pompidou, Paris, France; 2 Service d'Ophtalmologie, ORL et Chirurgie Cervico-faciale. Hôpital Robert Debré, Paris, France; 3 Université de Paris V, Paris, France; The University of Western Ontario, Canada

## Abstract

The purpose of this study was to test postural control during quiet standing in nonspecific chronic low back pain (LBP) subjects with vertical heterophoria (VH) before and after cancellation of VH; also to compare with healthy subjects with, and without VH. Fourteen subjects with LBP took part in this study. The postural performance was measured through the center of pressure displacements with a force platform while the subjects fixated on a target placed at either 40 or 200 cm, before and after VH cancellation with an appropriate prism. Their postural performance was compared to that of 14 healthy subjects with VH and 12 without VH (i.e. vertical orthophoria) studied previously in similar conditions. For LBP subjects, cancellation of VH with a prism improved postural performance. With respect to control subjects (with or without VH), the variance of speed of the center of pressure was higher, suggesting more energy was needed to stabilize their posture in quiet upright stance. Similarly to controls, LBP subjects showed higher postural sway when they were looking at a target at a far distance than at a close distance. The most important finding is that LBP subjects with VH can improve their performance after prism-cancellation of their VH. We suggest that VH reflects mild conflict between sensory and motor inputs involved in postural control i.e. a non optimal integration of the various signals. This could affect the performance of postural control and perhaps lead to pain. Nonspecific chronic back pain may results from such prolonged conflict.

## Introduction

Back pain is a common concern for many people and is a major public health problem. About a third of all back pain risks becoming chronic back pain [1], [2]. It is the most common chronic illness before the age of 65 [3], [4] and has a real and significant economic impact in industrialized countries [2], [4]–[8]. Among back pains, chronic low back pain (LBP) is the most frequent, even more so than chronic neck pain, and its prevalence is around 23% [9]–[11].

There is a simple and practical classification that has gained international acceptance, which divides LBP into three categories [11], [12]: i) specific spinal pathology, for instance infection, tumors, fractures or rheumatic diseases; ii) nerve root pain/radicular pain; iii) nonspecific LBP, i.e. not attributable to a recognizable known specific pathology. This last category represents up to 85% of people suffering from back pain [13].

Postural disorders are often taken into consideration in back pain; notably, a degradation of balance control in upright stance, evaluated with a force platform, is prevalent in LBP [e.g. 14–18]. Vertical heterophoria (VH) and vertical orthophoria (VO) are respectively the presence or the absence of a relative deviation of the vertical visual axes when the retinal pictures are dissociated, reduced via binocular vision mechanisms [19], [20]. VH exists in normal subjects, inferior to 1 diopter, on average 0.16±0.01° corresponding to 0.28 diopter [21]. Amos and Rutstein [19], and Scheiman and Wick [22], reported that subjects with VH present various complaints such as back pain. Clinical study of the management of nonspecific chronic pain suggested an association with VH and balance problems that were clinically evaluated [23]. Indeed, Matheron et al. [23] reported that in patients with nonspecific chronic pain associated with VH, a specific proprioceptive physiotherapy acting on oropharynx, temporomandibular joint and/or pelvis most of the time restored VO immediately (see [24]), diminished pain (evaluated with a subjective visual analog scale – VAS – [25], [26]), improved mobility of spinal and peripheral joints, and normalized behavior in the balance tests after initial alternation, but remain to be precisely evaluated. During controlled experiments, when VH was artificially induced in healthy subjects by the insertion of a small vertical prism (about 1°) during quiet standing, the postural control/performance was modified [27]. In normal subjects, Huang and Ciuffreda [28] showed that stronger yoked prisms (about 10°) induced a discrepancy between subjective and objective egocentric spaces, and then due to adaptation, such discrepancy rapidly declined. Such prisms are also used for postural problems in optometry/neuro-optometry and rehabilitation. For instance, they are used to reduce abnormal egocentric localization in individuals with brain injury, especially in traumatic brain injury and after cerebrovascular accidents (e.g. [29]–[33]). Additionally, the use of small prisms are known to induce postural behavior change in healthy subjects [27], [34]. In different postural disorders, they can improve balance, posture, and decrease subjective complaints (e.g.[35]–[37]), for instance in the case of vertigo [38], [39], in post-commotional syndrome following traumatic brain injury [39], [40] or in other sensory integration dysfunction [41]. Previous studies showed that young healthy adults with VH: i) showed lower postural performance in quiet upright stance than those with VO, particularly when they looked at a target placed at a farther distance; ii) improved their postural performance when VH was canceled with an appropriate vertical prism [42].

The aim of this study was to test postural control during quiet standing in nonspecific chronic LBP young adults with VH fixating on a target at a far or near distance before and after cancellation of VH. Then, we compared their natural postural performance with those of the healthy young adults observed in the previous study of Matheron and Kapoula [42] with and without VH.

In nonspecific chronic LBP subjects, the results showed that: cancellation of VH with a prism improved postural performance; their spontaneous postural control required more energy to stabilize their posture in the quiet upright stance compared to healthy subjects (with or without VH).

## Materials and Methods

### Ethics Statement

The postural control investigation adhered to the tenets of the Declaration of Helsinki and was approved by the local human experimentation committee, the “Comité de Protection des Personnes” (CPP) Ile de France VI (No: 07035), Necker Hospital, in Paris. Written informed consent was obtained from all subjects after the nature of the procedure was explained.

### Subjects

Fourteen nonspecific chronic LBP subjects were included in the present experimentation: 7 females and 7 males in the age range of 15–32 years (mean age  =  25.7±5.1 years, mean height =  171.4±8.6 cm, mean body weight =  63.0±6.4 kg) who were recruited from clinical centers. Inclusion criteria for these young adult subjects were the following: medical consultation and complementary examination (radiographic imaging as plain radiography, bone scanning or magnetic resonance imaging, and other tests such as blood analysis) did not report anatomical findings, neuropathy or rheumatism, nor repetitive traumatisms, but reported a nonspecific chronic LBP [11], [12] lasting longer than 6 months according to the World Health Organization criteria [43]; not undergoing treatment, neither physical, nor chemical (i.e. not in acute pain); and none of the subjects wore glasses, in order to avoid all prismatic effects, and thus vertical eye misalignment [19], [44], [45].

Comorbidity in nonspecific chronic LBP patients is common [46]–[48], and the other complaints were listed. Results are shown in Table 1. Pain in general was evaluated using a subjective visual analogical scale of 10 cm (VAS [24]); VAS has been validated for chronic pain [26].

**Table 1:** Different complaints for all nonspecific chronic LBP subjects.

Location of pain (%)	Other subjective complaints (%)
Lower back (100)	Tinnitus (36)
Middle back (43)	Visual strain (57)
Neck (71)	Dizziness (50)
Lower limbs (71)	Clumsiness gestural (50)
Upper limbs (57)	Vasomotor disturbances (36)
Headache (57)	VAS of pain (4.41±0.92)
TMJ (43)	
Eyes (36)	
Abdomen (36)	

Results are indicated in percent (location of pain, and other subjective complaints), and the mean and standard deviations evaluated with the VAS on which « 0 » indicated no pain and « 10 » the maximum amount of pain. (TMJ: temporomandibular joint).

The Maddox Rod Test, which is one of the most appropriate tests for clinically detecting the vertical heterophoria [45], [49], [50], was used, and combined with the bar-prism to measure the deviation of the eyes [20]. We found VH inferior to one diopter (i.e. in physiological range [21]) in all our subjects; this is in line with a previous clinical study reporting that a small amount of VH exists in 99% of 563 subjects suffering from nonspecific chronic pain [51]. In order to measure VH more accurately, the following procedure was used. First, the Maddox Rod Test was carried out for each subject for both eyes; a small prism (0.25, 0.50 or 0.75 diopter) was placed over the eye to cancel the VH. The value of the prism used corresponded to the value of the VH. Secondly, prisms were placed on each eye in turn and we retained the final prism correction, the prism that eliminated vertical heterophoria when the Maddox Rod Test was carried out on each eye. The final prism was used for postural recording conditions (see below). For more details, see the study of Matheron and Kapoula [42] where the same procedure was used. Results are shown in Table 2.

**Table 2:** Detection and measurement of the vertical phoria.

	Vertical phoria		Prism correction		
Subjects	RE	LE	Amount	Orientation	Eye
S1	HP 0.25 Δ	HR 0.25 Δ	0.25 Δ	BU	RE
S2	HP 0.25 Δ	HR 0.25 Δ	0.75 Δ	BD	LE
S3	VO	HP 0.25 Δ	0.25 Δ	BU	LE
S4	HP 0.25 Δ	VO	0.25 Δ	BU	RE
S5	HP 0.25 Δ	VO	0.25 Δ	BD	LE
S6	VO	HP 0.25 Δ	0.25 Δ	BU	RE
S7	VO	HR 0.25 Δ	0.25 Δ	BU	RE
S8	HR 0.25 Δ	HP 0.25 Δ	0.25 Δ	BU	RE
S9	HR 0.25 Δ	HR 0.25 Δ	0.25 Δ	BD	RE
S10	HR 0.25 Δ	HR 0.25 Δ	0.25 Δ	BU	LE
S11	HP 0.50 Δ	HR 0.50 Δ	0.50 Δ	BD	RE
S12	HR 0.25 Δ	HP 0.25 Δ	0.25 Δ	BD	RE
S13	HR 0.25 Δ	VO	0.25 Δ	BU	LE
S14	HP 0.25 Δ	HR 0.50 Δ	0.25 Δ	BD	LE

For the right eye (RE) and the left eye (LE) using the Maddox Rod Test in 14 subjects: vertical orthophoria (VO), hyperphoria (HR) and hypophoria (HP) which are vertical heterophoria (VH), respectively upward and downward deviation; and their prismatic correction: the amount (in diopter), orientation of the vertical prism - base up (BU) or base down (BD) - and the eye that received it (RE or LE) for the subject viewing was VO on both the left and the right.

### Platform characteristics

Postural performance during quiet standing was investigated through the center of pressure (CoP) displacements recorded using a force platform (principle of strain gauge) consisting of two dynamometric clogs (Standards by Association Française de Posturologie; produced by TechnoConcept, Céreste, France). The excursions of the CoP were measured over a period of 25.6 s; the equipment contained an Analog–Digital converter of 16 bits and the sampling frequency of the CoP was 40 Hz.

### Visual target

A vertical screen was used to display a target along the vertical midline. The target was a letter “x” placed between two vertical segments. The angular size of the letter “x” was adjusted to subtend 1° for both viewing distances (200 and 40 cm). At 200 cm, the angle of vergence was 2° while at 40 cm it was 9°. The visual target was placed at eye level for each subject in upright stance on the force platform.

### Testing conditions

**Figure 1:**
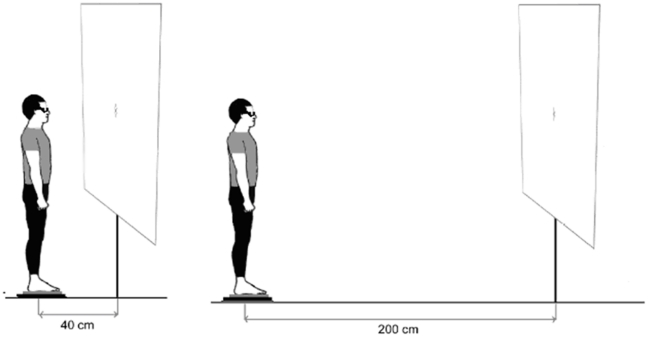
Illustrations of posturography testing conditions. The subject viewed a cross target embedded by two vertical line segments that aimed to reinforce accurate fixation of the letter ‘‘x’’, at 40 cm and 200 cm.

Quiet stance posturography was carried out in an experimental room which was furnished normally. The subjects wore a special spectacle upon which one could easily insert or not a vertical prism, and were placed barefoot on the force platform. They stayed under quiet upright and standardized position (feet placed side by side, forming a 30° angle with heels separated 4 cm). They were asked to fixate on the ‘‘x’’ target in the straight ahead position with or without the prism correction, i.e. in VO or VH, respectively. The target was placed at either 200 cm or 40 cm, at eye level (see Fig. 1). During posturography, subjects looked at the target that was clearly visible for both distance conditions. The order of the two distances was counterbalanced between subjects. For each distance, each testing condition (with and without prism) over the period of 25.6 s was done twice and was counterbalanced, i.e. for each distance, four counterbalanced recordings. A one-minute rest period was applied between any two conditions, at the beginning of this period the prism was inserted or removed. This procedure was previously applied to investigate postural control in healthy young adults with a natural VH where vertical prisms were used to cancel it [42]. When small prisms are inserted or taken out, eye movement response and binocular fusion are known to occur within a few seconds (e.g. [52]–[56]). In another study, Matheron et al. [27] induced an experimental VH with a two-diopter vertical prism; a one-minute rest period was applied between any two randomized conditions (no prism, prism over the dominant eye, and prism over the non-dominant eye). Postural stability improved when the vertical prism was inserted in front of the dominant eye, but decreased when it was inserted in front of the non-dominant eye. From a methodology point of view, this study indicated that one minute rest period between conditions is sufficient to override the prism effect due to prior condition.

### Postural parameters

We analyzed the surface of CoP excursions, the standard deviations of lateral (SDx) and antero-posterior (SDy) of the CoP and its variance of speed. The surface area was measured with the confidence ellipse including 90% of the CoP positions sampled [57], [58], eliminating the extreme points.

### Statistical analysis

For each distance, the data for the same conditions was averaged.

A mixed ANOVA design was used with two main factors: the distance with two levels – 40 cm and 200 cm –; and the vertical phoria with two levels – VH, and with the prism correction (PC) to cancel it –. The post hoc comparisons were done by the Scheffé post hoc test; *p*<0.05 was considered significant.

## Results

**Figure 2:**
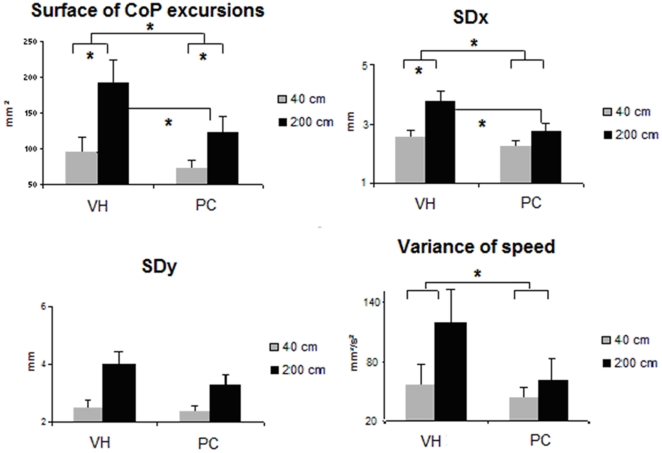
Effects of vertical phoria conditions and distance conditions in LBP subjects on postural parameters. Means of the surface of CoP excursions (mm^2^), the standard deviation of lateral (SDx) and antero-posterior (SDy) postural sways (mm), and the variance of speed of CoP (mm^2^/s^2^) in vertical heterophoria (VH) condition and after prism correction (PC) for each distance (40 cm and 200 cm). Error bars represent the standard error. Asterisks indicate significant differences.

The results of ANOVA evaluating the effects of vertical phoria conditions and distance conditions in nonspecific chronic LBP young adults with VH on postural parameters, i.e. the surface area of the CoP excursions, SDx, SDy, and the variance of speed of the CoP (see Fig. 2), were the following:

### Distance effect

Except on the variance of speed (F_(1,13)_ = 0.61; *p*>0.05), there was a main effect of distance on all other postural parameters tested: the surface of CoP excursions (F_(1,13)_ = 25.46; *p* = 0.0002), SDx (F_(1,13)_ = 13.46; *p* = 0.003), SDy (F_(1,13)_ = 18.27; *p* = 0.0009). All these parameters were significantly smaller at a close distance than at a far distance.

### Vertical phoria effect

There was no main effect on SDy (F_(1,13)_ = 2.62; *p*>0.05), but a significant main vertical phoria effect on the surface of CoP excursions (F_(1,13)_ = 7.64; *p* = 0.016), on SDx (F_(1,13)_ = 11.23; *p* = 0.005), and on the variance of speed of the CoP (F_(1,13)_ = 13.83; *p* = 0.003) where these parameters were significantly higher in VH condition than in the prism correction condition, i.e. when VH was canceled.

### Interaction between the vertical phoria and the viewing distance

There was a significant interaction between the vertical phoria condition and the viewing distance for the surface of CoP excursions (F_(1,13)_ = 4.68; *p*<0.05) and a tendency for SDx (F_(1,13)_ = 4.47; *p* = 0.054).

For the local comparisons between distances, the Scheffé post hoc test showed that the surface of CoP excursions and SDx were significantly smaller at the closer viewing distance than the father one in VH condition (*p* = 0.0003 and *p* = 0.002, respectively), and after the prism correction on the surface of CoP (*p* = 0.044).

For the local comparisons between the vertical phoria and the viewing distance, there was no difference before and after prism correction at 40 cm, but the Scheffé post hoc test showed a significant difference where the surface of CoP excursions and SDx were significantly smaller at 200 cm after the prism correction (*p* = 0.005 and *p* = 0.007, respectively).

In all, the results showed main effects (i) of distance, i.e. increasing of postural sway when the distance increased, (ii) of vertical phoria where the postural sway decreased in prism correction condition, i.e. when VH is cancelled, notably when subjects are looking at a target at a far distance.

#### Results for postural control in chronic LBP vs. healthy adults with and without VH

In a previous study, Matheron and Kapoula [42] studied the postural control during quiet standing in 26 healthy young adults (15 females, 11 males) in the age range of 22–34 years (27.04±3.29 years) with VH vs. no VH (i.e. VO) with exactly the same experimental setup (posturography, number of trials, distances, targets). Subjects with VH showed greater instability than subjects without VH. Here, we compared postural performances of both healthy subject groups without nonspecific chronic LBP, i.e. the healthy group with VH and the healthy group with VO vs. those in our present chronic LBP subjects with the same postural parameters (i.e. the surface of CoP excursions, SDx, SDy, and the variance of speed of the CoP). Both healthy groups were composed of the following: 1) Fourteen healthy subjects with VH (HSVH): 7 females and 7 males in the age range of 22–31 years (mean age  = 26.6±2.9 years, mean height  =  170.7±8.4 cm, mean body weight  =  64.1±9.2 kg); 2) Twelve healthy subjects with VO (HSVO): 8 females and 4 males in the age range of 22–34 years (mean age  =  27.6±3.7 years, mean height  =  167.5±8.2 cm, mean body weight =  59.2±8.5 kg). Between the three groups (LBP, HSVH, HSVO), there was no statistically significant difference in terms of age, sex, height and weight. After applying the homogeneity test, the postural data were subjected to a mixed ANOVA design with the viewing distance as main factor with two levels (40 cm and 200 cm), and one inter-subject factor with three levels (nonspecific chronic LBP, HSVH, HSVO). Post hoc analyses (Scheffé tests) were used when appropriate. The level of significance was always set at *p*<0.05.

**Figure 3:**
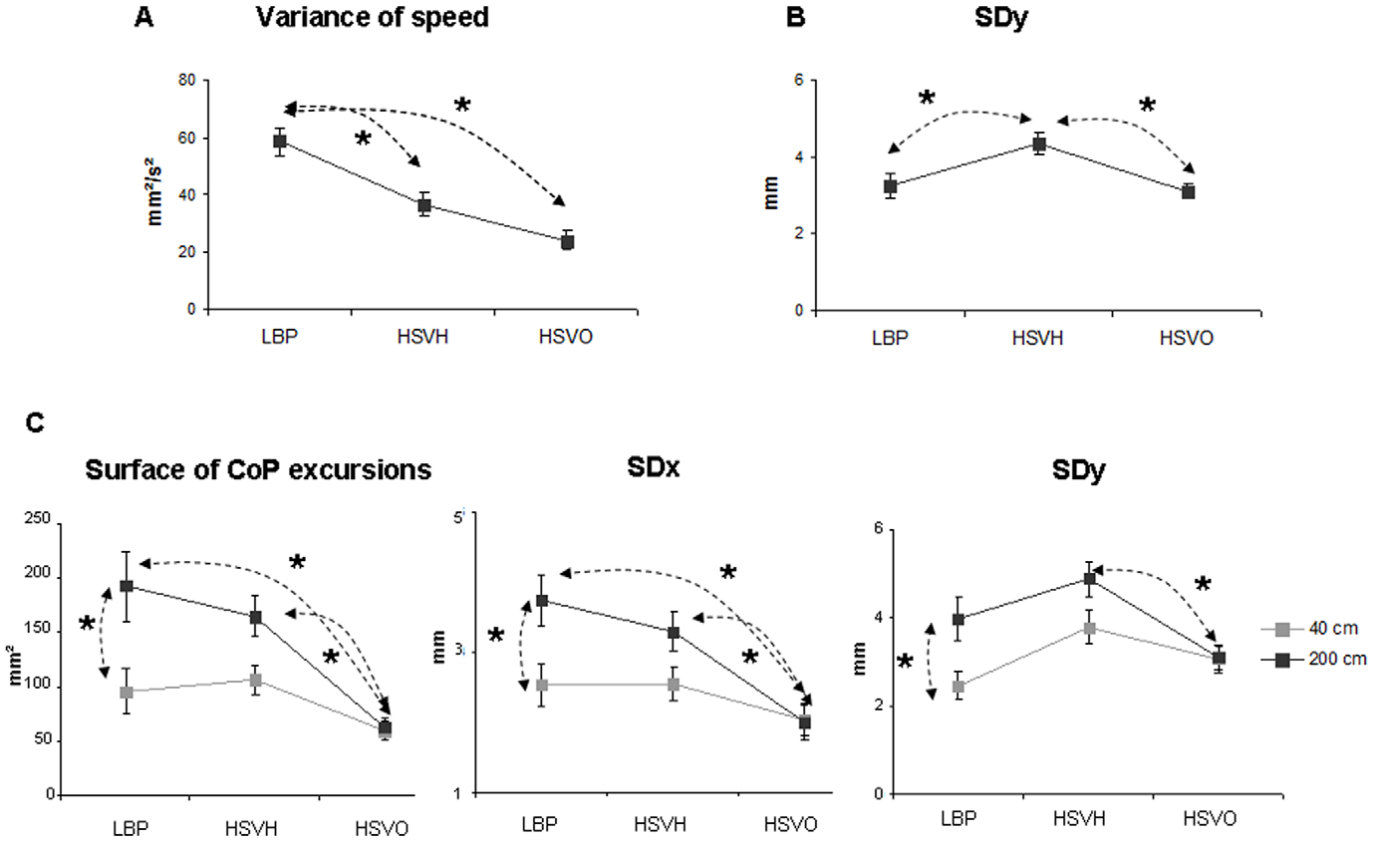
Group mean values of each postural parameter. Means of the variance of speed of CoP (mm^2^/s^2^) (**A**) and the antero-posterior (SDy) postural sways (mm) (**B**) in nonspecific chronic low back pain subjects (LBP), and in healthy subjects with vertical heterophoria (HSVH) and without VH, i.e. with vertical orthophoria (HSVO) – data for the distance are grouped. Means of the surface of CoP excursions (mm^2^), the standard deviation of lateral (SDx) and antero-posterior (SDy) postural sways (mm) (**C**) in LBP, HSVH and HSVO for each distance (40 cm and 200 cm). Error bars represent the standard error. Asterisks indicate significant differences.

Beside the main effect of distance found again (not shown here), the results were the following (see Fig. 3 for main results):

### Group effect

There was a group effect on all parameters studied: the surface of CoP excursions (F_(2,37)_ = 6.54; *p* = 0.004), SDx (F_(2,37)_ = 5.31; *p* = 0.009), SDy (F_(2,37)_ = 5.12; *p* = 0.01), and the variance of speed of the CoP (F_(2,37)_ = 10.11; *p* = 0.0003). The Scheffé post hoc test showed that the variance of speed of the CoP was significantly higher in nonspecific chronic LBP than in HSVH (*p* = 0.02) and than in HSVO (*p* = 0.0004) – see Fig. 3a. For other parameters, the post hoc revealed higher values: for the surface of CoP in chronic LBP and HSVH compared to HSVO (respectively *p* = 0.008, *p* = 0.02), for SDx in nonspecific chronic LBP compared to HSVO (*p* = 0.01), and for SDy in HSVH compared to nonspecific chronic LBP (*p* = 0.04) and to HSVO (*p* = 0.03) – see Fig. 3b.

### Interaction between groups and the viewing distance

There was no interaction between the group and the viewing distance for the variance of speed (F_(2,37)_ = 0.06; *p*>0.05). Conversely, there was a significant interaction for the surface of CoP excursions (F_(2,37)_ = 6.33; *p* = 0.004), for SDx (F_(2,37)_ = 4.96; *p* = 0.01), and a tendency for SDy (F_(2,37)_ = 2.90; *p* = 0.06) – see Fig. 3c.

For the local comparisons between distances, the Scheffé post hoc test showed that the surface of CoP excursions, SDx and SDy were significantly higher at 200 cm than at 40 cm for the group of subjects suffering from nonspecific chronic LBP (*p* = 0.0005, *p* = 0.005 and *p* = 0.04, respectively).

For the local comparisons between the group and the viewing distance, there was no difference between groups at 40 cm, but the Scheffé post hoc test showed a significant difference at 200 cm where the surface of CoP excursions and SDx were significantly higher in the group of subjects suffering from nonspecific chronic LBP (*p* = 0.000006 and *p* = 0.00007, respectively) and in HSVH (*p* = 0.0004 and *p* = 0.005, respectively) than in HSVO. When at the farther distance, SDy was significantly higher in HSVH than in HSVO (*p* = 0.02).

Finally, besides the main effect of distance where postural sway increased with distance, with a stronger effect in chronic LBP subjects than in subjects without LBP, the results showed (i) the main effect of the group where the variance of speed of the CoP was higher in chronic LBP subjects, (ii) that antero-posterior postural sway was lower in chronic LBP subjects than in healthy subjects with vertical heterophoria.

## Discussion

All the young adults suffering from a nonspecific chronic LBP [11], [12] for more than 6 months according to the World Health Organization criteria [43] included in the present experimentation reported comorbid subjective heath complaints as in other studies [46]–[48]; if the dominant complaint was at the low back level, it was systematically with other symptoms, notably in the two-thirds of these cases at the cervical spine and the lower limb levels. On the other hand, all these subjects had a VH as indicated in a previous clinical study concerning the chronic pain aspect [51]; VH was in the range of physiological value, i.e. inferior to one diopter [21].

### In chronic LBP subjects, the cancellation of VH with a prism improved postural performance

The first important point is that in young adults suffering from nonspecific chronic LBP, the link previously described by Matheron and Kapoula [42] between vertical phoria and postural control in undisturbed quiet upright stance was found again. Indeed, clearly the cancellation of VH with an appropriate prism, i.e. to obtain VO for both eyes when the Maddox Rod Test was run, improved the postural performance; this is in line with the results of the study by Matheron and Kapoula [42] in which healthy young adults with VH showed higher postural sway than those with VO. VH could affect the postural control via neuroanatomic connections between the cerebellum and circuits of proprioceptive signals (see [42]) such those from extraocular muscles on the one hand, and oculomotor circuits on the other hand, the cerebellum controlling both eye movements and bearing of binocular alignment [59], [60], and a stable upright standing [61]. Of course, this result with the cancellation of VH did not show nor prove a link between VH and chronic LBP, but it was in line with clinical studies reporting that in nonspecific chronic pain subjects associated with VH, a specific proprioceptive physiotherapy applied to dysfunctional levels most of the time restored VO immediately, improved clinical balance tests and diminished pain intensity [23], [24]. Low limb and trunk proprioceptive signals are important in balance control and posture [62]–[64]; spine muscles are both a sensory captor and a motor effector [18]. Patients with low back pain showed a reduced lumbosacral proprioception which might lead to a decrease in their postural performance compared to healthy subjects [17], [18], [65], [66]; this alteration could explain an underlying dysfunction of the peripheral proprioceptive sensor, the central integration of the proprioceptive signal [18], [66], [67], or muscular efficiency [68]. In this context, the results showing a significant improvement in the postural performance through CoP displacements of chronic LBP subjects when their VH were canceled reinforce previous expectations: VH, even when small in size, could indicate a perturbation of the somatosensory/proprioceptive loops involved in postural control [27]. We suggest that VH could reflect a mild global sensorimotor conflict between sensory and motor inputs i.e. a non optimal integration of the various signals. Poor integration of somesthetic cues could affect the performance of balance control and lead to pain. This speculation is in line with the experimental model introduced by McCabe et al. [69], [70] providing evidence that sensorimotor conflict (between vision and proprioceptive cues) can induce pain and modify sensory perception in some normal subjects, and suggested that it could lead to long term symptoms if prolonged. We hypothesize that nonspecific chronic back pain could result from such prolonged conflict. In other words, in the absence of neurological, visual or ocular disease, vertical heterophoria could be a sign of a pre-existing sensory-motor conflict, as proposed in fibromyalgia [70], here implicating somesthetic cues. Such prolonged conflict could lead to pain, chronic pain and associated symptomatic comorbidity if compensatory mechanisms become unable to solve the conflict; perhaps after an undetermined precipitating event, such as physical effort, a repeated movement at work or while playing sports which can factor into compensatory limitations. Moreover, Harris [71] suggested that sensory conflict inputs to the central nervous system could lead to unpleasant sensations and chronic pain; and recently in a retrospective analysis, Doble et al. [72] identified such VH in traumatic brain injury patients and reported improvement of effects of individualized prismatic spectacle lenses to correct it in the treatment of postconcussive symptoms as dizziness and back pain.

### Chronic LBP subjects used more energy to stabilize postural sway during quiet standing

A group effect was found on the variance of speed of CoP displacements: thus, nonspecific chronic LBP appears to affect the dynamic of excursions of CoP on this parameter in undisturbed quiet upright stance. Interestingly, the cancellation of the VH with an appropriate prism in nonspecific chronic LBP subjects decreased the variance of speed; such effect was not found after the cancellation of the VH in healthy subjects [42] or when experimental VH was induced in healthy subjects [27]. This observation is in line with our previously mentioned expectation of prolonged conflict. Specific increasing of the variance of speed at whatever distance the target fixation at eye level, suggests that chronic LBP subjects used more low limb muscles than healthy subjects to maintain balance in upright stance; this could reflect the need of more energy to stabilize postural sway. It was believed that variance of speed of CoP displacement is related to the energy used to achieve postural stabilization, namely, leg muscle activity [73]–[75]. Indeed, the variance of speed indicates the dispersion of the mean speed of the pressure. High speed variance suggests increased variance of foot pressure which is related to leg activity: the link between the shift of CoP and leg group muscle activity has been shown by Wang et al. [73], and was consistent with studies from Amiridis et al. [76] and Jonsson et al. [77] who reported increased muscle activity in aged subjects in order to stabilize posture.

### Nonspecific chronic LBP subjects exhibited lower antero-posterior postural sway than in healthy subjects with vertical heterophoria

There was a significant group effect on the standard deviations of antero-posterior excursions of the CoP where they were lower in nonspecific chronic LBP subjects than in healthy subjects with vertical heterophoria. These results differ from those of Hamaoui et al. [16] who found that antero-posterior CoP displacements were largest in chronic LBP subjects tested in static posturography, whose feet were close together. But, these authors did not find this difference with feet spread in the vertical projection of the hips, a similar position to the one analyzed in this study; note also that when we compared all healthy subjects (i.e. those with VH plus VO) vs. nonspecific chronic LBP subjects, we did not find any difference (F_(1,38)_ = 1.74; *p*>0.05 – not shown here). Other studies found that patients suffering from low back pain showed larger oscillations than controls when an external perturbation occurred, or in dynamic conditions, but did not find any difference in undisturbed quiet upright stance [17], [67], [78]–[80]. Now, when the vertical phoria status was taken into account in the control group, there was a significant change in postural behavior, i.e. the amplitude of anteror-posterior body oscillation through CoP excursions was lower in nonspecific chronic LBP subjects than in healthy subjects with vertical heterophoria. This difference could be linked to a change in the strategy of postural control in patients. Brumagne et al. [66], [80] and Popa et al. [81] reported that in back pain, patients stiffened the trunk, the pelvis and the low limbs to decrease the number of degrees of freedom, leading to tighter control of CoP excursions and so, smaller postural sways. Moreover, Stokes et al. [82] with electromyography showed that before a transient force perturbation, subjects with back pain had a greater muscle preactivation than control subjects. So, increased variance of speed, which we previously discussed, and lower antero-posterior CoP displacements in our chronic LBP subjects are coherent observations illustrating revealing the specific strategy used to stabilize postural sway. Furthermore, Mazzocchio et al. [83], [84] showed in electrophysiological studies the largest muscle activity in the legs during their upright stance. On the other hand, according to Popa et al. [81], this motor strategy, i.e. stiffness and increased muscle activity, could be due to reduced accuracy in the sensory integration process of proprioceptive cues; we share the hypothesis of reduced integration as discussed here and in our prior studies (see [42]). Further studies are needed to test it.

### Chronic LBP subjects' postural performance decreased when distance fixation increased

Distance effect was found on postural control in chronic LBP subjects as in healthy subjects. Previously in healthy subjects, an interaction between vertical phoria and distance where the subjects with VH showed greater instability than the subjects with VO at a far distance was described; an additional study showed that the cancellation of VH with a prism improved postural stability [42]. The present study provides similar distance effects in nonspecific chronic LBP subjects. Nevertheless, an interaction was found indicating for the far distance larger surface of CoP, larger SDx and SDy for LBP patients (see Fig. 3c). During quiet upright stance, visual stabilization of posture decreases when the target fixation distance increases, which is attributed to decreased angular size of retinal slip induced by body sway [85]–[88] and ocular motor signals from the converging eyes involved at a close distance [74], [75].

To our knowledge, it was the first time that postural control when fixating a viewing target at different distances was investigated in subjects suffering from back pain. Kapoula and Lê [74] showed that distance effect was linked to decreasing ocular motor signals from eye convergence angle: postural sway increased as distance target fixation increased. The authors suggested that beyond 90 cm, the central nervous system would use mostly internal signals, i.e. vestibular and somesthetic as proprioceptive cues [75]. Now, low limb and spine proprioceptive afferences are required in balance and postural control in upright stance [62]–[64]; in low back pain there is a local proprioceptive deficit [17], [18], [65], [66] which could disturb signal integration [18], [67] and lead to less performance in postural control when the target is placed at a far distance. Furthermore, to compensate for the proprioceptive deficit, the central nervous system would increase reliance on vision in the postural control in chronic LBP subjects [17], [67], [78], [89], the weight of vision [85]–[88], [90] and oculomotor signals [74], [75] which decreases with distance. Thus, the far condition better illustrates the hypothetical proprioceptive deficit in such patients.

In conclusion, this study confirms the following: i) small size VH exists in nonspecific chronic pain as mentioned in a previous clinical study [51]; ii) the link between VH and postural control in undisturbed quiet upright stance. Indeed in nonspecific chronic LBP subjects, the VH cancellation with an appropriate vertical prism significantly improved postural performance as it did in healthy subjects with similar VH [42]. Nonspecific chronic LBP subjects used more energy than healthy subjects to achieve postural sway stabilization when looking at a fixed target no matter the distance, and their CoP displacements increased in far vision. This suggests the interest, in both a clinical and an experimental context, to carry out static posturography recordings at a far distance, at least in this pathology. The hypothesis speculating that VH, even when small in size as far as considered as physiological, could indicate that a perturbation of the sensorimotor loops involved in postural control [42] is reinforced; further studies are necessary to verify if VH is an integrative weakness or simply a higher threshold of imperfection in eye alignment tolerated by the central nervous system. In back pain, Della Volpe et al. [67] and Missaoui et al. [18] suggested that a dysfunction implicating somesthetic signals or central neurological integration could exist and affect the balance control performance. We suggest that VH could be a sign of such dysfunction, perhaps related to cerebellum receiving both visual and proprioceptive signals and controlling both eye alignment and posture. VH could reflect a mild global sensorimotor conflict between sensory, such as somesthetic, and motor inputs affecting the performance of balance control and maybe lead to pain. Perhaps nonspecific chronic back pain results from such prolonged conflict.

## References

[1] Croft PR, Macfarlane GJ, Papageorgiou AC, Thomas E, Silman AJ (1998). Outcome of low back pain in general practice: a prospective
study.. BMJ.

[2] Thomas E, Silman AJ, Croft PR, Papageorgiou AC, Jayson MI (1999). Predicting who develops chronic low back pain in primary care: a
prospective study.. BMJ.

[3] Waddell G (1996). Low back pain: a twentieth century health care
enigma.. Spine.

[4] Andersson GBJ (1999). Epidemiological features of chronic low-back
pain.. Lancet.

[5] Cats-Baril WL, Frymoyer JW (1991). Identifying patients at risk of becoming disabled because of
low-back pain. TheVermont Rehabilitation Engineering Center predictive
model.. Spine.

[6] Waddell G (1993). Simple low back pain: rest or active exercice?. Ann Rheum Dis.

[7] Maniadakis N, Gray A (2000). The economic burden of back pain in the UK.. Pain.

[8] Atlas SJ, Nardin RA (2003). Evaluation and treatment of low back pain: An evidence-based
approach to clinical care.. Muscle Nerve.

[9] Andersson HI, Ejlertsson G, Leden I, Rosenberg C (1993). Chronic pain in a geographically defined general population:
studies of differences in age, gender, social class, and pain
localization.. Clin J Pain.

[10] Walker BF (2000). The prevalence of low back pain: a systematic review of the
literature from 1966 to 1998.. J Spinal Disord.

[11] European guidelines for the management of low back
pain (2004). http://www.backpaineurope.org/web/files/WG2_Guidelines.pdf.

[12] Waddell G (1987). A new clinical model for the treatment of low-back
pain.. Spine.

[13] Deyo RA (1988). Measuring the functional status of patients with low back
pain.. Arch Phys Med Rehabil.

[14] Byl NN, Sinnot P (1988). Variations in balance and body sway in middleaged adults.
Subjects with healthy backs compared with subjects with low-back
dysfunction.. Spine.

[15] Alexander KM, LaPier TL (1998). Differences in static balance and weight distribution between
normal subjects and subjects with chronic unilateral low back
pain.. J Orthop Sports Phys Ther.

[16] Hamaoui A, Do MC, Bouisset S (2004). Postural sway increase in low back pain subjects is not related
to reduced spine range of motion.. Neurosci Lett.

[17] Mok NW, Brauer SG, Hodges PW (2004). Hip strategy for balance control in quiet standing is reduced in
people with low back pain.. Spine.

[18] Missaoui B, Portero P, Bendaya S, Hanktie O, Thoumie P (2008). Posture and equilibrium in orthopedic and rheumatologic
diseases.. Neurophysiol Clin.

[19] Amos FJ, Rutstein RP, Amos FJ (1987). Vertical deviation.. Diagnosis and management in vision care.

[20] von Noorden GK (1996). Binocular vision and ocular motility: theory and management of
strabismus..

[21] van Rijn U, ten Tusscher MP, de Jong I, Hendrikse F (1998). Asymmetrical vertical phorias indicating dissociated vertical
deviation in subjects with normal binocular vision.. Vision Res.

[22] Scheiman M, Wick B (1994). Clinical management of binocular vision, heterophoric,
accommodative and eye movement disorders..

[23] Matheron E, Quercia P, Weber B, Gagey PM (2005). Vertical heterophoria and postural deficiency
syndrome.. Gait Posture.

[24] Matheron E (2000). [Vertical heterophoria and myotonic
normalisation].. Kinésithér Scient.

[25] Huskisson EO (1974). Measurement of pain.. Lancet.

[26] Price DD, McGrath PA, Rafii A, Buckingham B (1983). The validation of visual analogue scales as ratio scale measures
for chronic and experimental pain.. Pain.

[27] Matheron E, Le TT, Yang Q, Kapoula Z (2007). Effects of a two-diopter vertical prism on
posture.. Neurosci Lett.

[28] Huang MA, Ciuffreda KJ (2006). Short-term adaptation to vertical yoked prisms.. Optom Vis Sci.

[29] Padula WV, Argyris S, Ray J (1994). Visual evoked potentials (VEP) evaluating treatment for
post-trauma vision syndrome (PTVS) in patients with traumatic brain injuries
(TBI).. Brain Inj.

[30] Kapoor N, Ciuffreda KJ (2002). Vision disturbances following traumatic brain
injury.. Curr Treat Options Neurol.

[31] Suchoff IB, Ciuffreda KJ (2004). A primer for the optometric management of unilateral spatial
inattention.. J Am Optom Assoc.

[32] Pisella L, Rode G, Farnè A, Tilikete C, Rossetti Y (2006). Prism adaptation in the rehabilitation of patients with
visuo-spatial cognitive disorders.. Curr Opin Neurol.

[33] Padula WV, Nelson CA, Padula WV, Benabib R, Yilmaz T (2009). Modifying postural adaptation following a CVA through prismatic
shift of visuo-spatial egocenter.. Brain Inj.

[34] Ushio N, Hinoki M, Nakanishi K, Baron JB (1980). [Role of ocular muscle proprioception in the maintenance of
body equilibrium with particular reference to the cervical
reflex].. Agressologie.

[35] Da Cunha HM, Da Silva OA (1986). [Postural deficiency syndrome. Its importance in
ophthalmology].. J Fr Ophtalmol.

[36] Gagey PM, Gentaz R, Bodot C (1987). [Postural checkup].. Agressologie.

[37] Marucchi C, Zamfirescu F, Gagey PM, Gentaz R, Guillaume P (1988). [Ophthalmologic determinants of prismatic correction of
posture. A retrospective analysis of 39 prescriptions].. Agressologie.

[38] Baron JB, Fowler E (1952). Prismatic lenses for vertigo and some experimental background of
the role of the extrinsic ocular muscles in disequilibrium.. Trans Am Acad Ophthal Oto-laryngol.

[39] Ushio N, Hinoki M, Baron JB, Takeya T, Grateau J (1980). [Prismatic therapy of vertigo and disequilibrium
particularly in patients with cranial and cranio-cervical
injuries].. Agressologie.

[40] Gagey PM, Bles W, Brandt Th (1980). Postural disorders among workers on building
sites.. Disorders of Posture and Gait.

[41] Allison CL, Gabriel H, Schlange D, Fredrickson S (2007). An optometric approach to patients with sensory integration
dysfunction.. Optometry.

[42] Matheron E, Kapoula Z (2008). Vertical phoria and postural control in upright stance in healthy
young subjects.. Clin Neurophysiol.

[43] Kaplun A, World Health Organization (1992). A new understanding chronic pain.. Health promotion and chronic illness.

[44] Scattergood KD, Brown MH, Guyton DL (1983). Artifacts introduced by spectacle lenses in the measurement of
strabismic deviations.. Am J Ophthalmol.

[45] Daum KM, Eskridge JB, Amos FJ, Barlett JD (1991). Heterophoria and heterotropia.. Clinical procedures in optometry.

[46] Hestbaek L, Leboeuf-Yde C, Manniche C (2003). Is low back pain part of a general health pattern or is it a
separate and distinctive entity? A critical literature review of comorbidity
with low back pain.. J Manipulative Physiol Ther.

[47] von Korff M, Crane P, Lane M, Miglioretti DL, Simon G (2003). Chronic spinal pain and physical-mental comorbidity in the United
States: results from the national comorbidity survey
replication.. Pain.

[48] Hagen EM, Svensen E, Eriksen HR, Ihlebaek CM, Ursin H (2006). Comorbid subjective health complaints in low back
pain.. Spine.

[49] Wong AM, Tweed D, Sharpe JA (2002). Vertical misalignment in unilateral sixth nerve
palsy.. Ophthalmology.

[50] Casillas Casillas E, Rosenfield M (2006). Comparison of subjective heterophoria testing with a phoropter
and trial frame.. Optom Vis Sci.

[51] Matheron E, Weber B, Villeneuve Ph (2007). Test de Maddox (stries verticales) et syndrome de
déficience posturale.. Posturologie clinique: dysfonctions motrices et cognitives.

[52] Kertesz AE, Schor CM, Ciuffreda KJ (1983). Vertical and cyclofusional disparity vergence.. Vergence Eye Movements.

[53] Ygge J, Zee DS (1995). Control of vertical eye alignment in three-dimensional
space.. Vision Res.

[54] Cheeseman EW, Guyton DL (1999). Vertical fusional vergence: the key to dissociated vertical
deviation.. Arch Ophthalmol.

[55] Matheron E, Yang Q, Le TT, Kapoula Z (2008). Effects of ocular dominance on the vertical vergence induced by a
2-diopter vertical prism during standing.. Neurosci Lett.

[56] Demer JL, Clark RA, Crane BT, Tian JR, Narasimhan A (2008). Functional anatomy of the extraocular muscles during
vergence.. Prog Brain Res.

[57] Takagi A, Fujimura E, Suehiro S, Igarashi M, Black O (1985). A new method of statokinesigram area measurement. Application of
a statistically calculated ellipse.. Vestibular and visual control on posture and locomotion
equilibrium.

[58] Gagey PM, Weber B, Masson (1999). Stabilométrie.. Posturologie: régulation et dérèglements de la
station debout.

[59] Kono R, Hasebe S, Ohtsuki H, Kashihara K, Shiro Y (2002). lmpaired vertical phoria adaptation in patients with cerebellar
dysfunction.. lnvest Ophthalmol Vis Sci.

[60] Sunartpin P, Kotchabhakdi N (2005). Afferent projections from motoneurons innervating extraocular
muscles to the cerebellum demonstrated by the retrograde double-labeling
technique.. J Med Assoc Thai.

[61] Diener HO, Dichgans J, Guschlbauer B, Bacher M, Langenbach P (1989). Disturbances of motor preparation in basal ganglia and cerebellar
disorders.. Prog Brain Res.

[62] Bergin PS, Bronstein AM, Murray NM, Sancovic S, Zeppenfeld DK (1995). Body sway and vibration perception thresholds in normal aging and
in patients with polyneuropathy.. J Neurol Neurosurg Psychiatry.

[63] Bloem BR, Allum JH, Carpenter MG, Honegger F (2000). Is lower leg proprioception essential for triggering human
automatic postural responses?. Exp Brain Res.

[64] Speers RA, Kuo AD, Horak FB (2002). Contributions of altered sensation and feedback responses to
changes in coordination of postural control due to aging.. Gait Posture.

[65] Brumagne S, Cordo P, Lysens R, Swinnen S, Verschueren S (2000). The role of paraspinal muscle spindles in lumbosacral position
sense in individuals with and without low back pain.. Spine.

[66] Brumagne S, Cordo P, Verschueren S (2004). Proprioceptive weighting changes in persons with low back pain
and elderly persons during upright standing.. Neurosci Lett.

[67] della Volpe R, Popa T, Ginanneschi F, Spidalieri R, Mazzocchio R (2006). Changes in coordination of postural control during dynamic stance
in chronic low back pain patients.. Gait Posture.

[68] Ebenbichler G, Oddsson L, Kollmitzer J, Erim Z (2001). Sensory-motor control of the lower back: implications for
rehabilitation.. Med Sci Sports Exer.

[69] McCabe CS, Haigh RC, Halligan PW, Blake DR (2005). Simulating sensory–motor incongruence in healthy
volunteers: implications for a cortical model of pain.. Rheumatology.

[70] McCabe CS, Cohen H, Blake DR (2007). Somesthetic disturbances in fibromyalgia are exaggerated by
sensory motor conflict: implications for chronicity of the
disease?. Rheumatology.

[71] Harris AJ (1999). Cortical origin of pathological pain.. The Lancet.

[72] Doble JE, Feinberg DL, Rosner MS, Rosner AJ (2010). Identification of binocular vision dysfunction (vertical
heterophoria) in traumatic brain injury patients and effects of
individualized prismatic spectacle lenses in the treatment of postconcussive
symptoms: a retrospective analysis.. PM R.

[73] Wang Y, Zatsiorsky VM, Latash ML (2006). Muscle synergies involved in preparation to a step made under the
self-paced and reaction time instructions.. Clin Neurophysiol.

[74] Kapoula Z, Lê TT (2006). Effects of distance and gaze position on postural stability in
young and old subjects.. Exp Brain Res.

[75] Lê TT, Kapoula Z (2006). Distance impairs postural stability only under binocular
viewing.. Vision Res.

[76] Amiridis IG, Hatzitaki V, Arabatzi F (2003). Age-induced modifications of static postural control in
humans.. Neurosci Lett.

[77] Jonsson E, Seiger A, Hirschfeld H (2005). Postural steadiness and weight distribution during tandem stance
in healthy young and elderly adults.. Clin Biomech.

[78] Mientjes MI, Frank JS (1999). Balance in chronic low back pain patients compared to healthy
people under various conditions in upright standing.. Clin Biomech.

[79] Henry SM, Hitt JR, Jones SL, Bunn JY (2006). Decreased limits of stability in response to postural
perturbations in subjects with low back pain.. Clin Biomech.

[80] Brumagne S, Janssens L, Janssens E, Goddyn L (2008). Altered postural control in anticipation of postural instability
in persons with recurrent low back pain.. Gait Posture.

[81] Popa T, Bonifazi M, Della Volpe R, Rossi A, Mazzocchio R (2007). Adaptive changes in postural strategy selection in chronic low
back pain.. Exp Brain Res.

[82] Stokes IA, Fox JR, Henry SM (2006). Trunk muscular activation patterns and responses to transient
force perturbation in persons with self-reported low back
pain.. Eur Spine J.

[83] Mazzocchio R, Scarfò GB, Cartolari R, Bolognini A, Mariottini A (1999). Abnormalities of the soleus H-reflex in lumbar spondylolisthesis:
a possible early sign of bilateral S1 root dysfunction.. J Spinal Disord.

[84] Mazzocchio R, Scarfo GB, Mariottini A, Muzii VF, Palma L (2001). Recruitment curve of the soleus H-reflex in chronic back pain and
lumbosacral radiculopathy.. BMC Musculoskelet Disord.

[85] Bles W, Kapteyn TS, Brandt T, Arnold F (1980). The mechanism of physiological height vertigo. II.
Posturography.. Acta Otolaryngol.

[86] Paulus WM, Straube A, Brandt T (1984). Visual stabilization of posture. Physiological stimulus
characteristics and clinical aspects.. Brain.

[87] Brandt T, Paulus W, Straube A, Bles W, Brandt T (1986). Vision and posture.. Disorders of posture.

[88] Paulus W, Staube A, Krafczyk S, Brandt T (1989). Differential effects of retinal targets displacement changing
size and changing disparity in the control of anterior/posterior and lateral
body sway.. Exp Brain Res.

[89] Jones KE, Wessberg J, Vallbo A (2001). Proprioceptive feedback is reduced during adaptation to a
visuomotor transformation: preliminary findings.. Neuroreport.

[90] Vuillerme N, Burdet C, Isableu B, Demetz S (2006). The magnitude of the effect of calf muscles fatigue on postural
control during bipedal quiet standing with vision depends on the
eye–visual target distance.. Gait Posture.

